# Tourniquet induced ischemia and changes in metabolism during TKA: a randomized study using microdialysis

**DOI:** 10.1186/s12891-015-0784-y

**Published:** 2015-10-29

**Authors:** Ashir Ejaz, Anders C. Laursen, Andreas Kappel, Thomas Jakobsen, Poul Torben Nielsen, Sten Rasmussen

**Affiliations:** Department of Orthopedic Surgery, Aalborg University Hospital, Aalborg, Denmark; Department of Clinical Medicine, Aalborg University, Aalborg, Denmark

**Keywords:** Microdialysis, Tourniquet, Ischemia, Metabolism, TKA, RCT

## Abstract

**Background:**

Tourniquet use in total knee arthroplasty (TKA) surgery is applied to minimize blood loss thereby creating better overview of the surgical field. This induces ischemia in the skeletal muscle resulting in reperfusion injury. Our aim was to investigate the in vivo metabolic changes in the skeletal muscle during TKA surgery using microdialysis (MD).

**Methods:**

Seventy patients were randomly allocated to tourniquet group (*n* = 35) or non-tourniquet group (*n* = 35). Prior to surgery, catheters were inserted in the operated leg and non-operated leg. Interstitial dialysate was collected before and after surgery and at 20 min intervals during a 5 h reperfusion period. Main variables were ischemic metabolites: glucose, pyruvate, lactate and glycerol and L/P ratio.

**Results:**

Significant difference in all metabolites was detected between the two groups, caused by tourniquet application. Tourniquet induced ischemia resulted in decreased levels of glucose and pyruvate to 54 and 60 % respectively, compared to baseline. Simultaneously, accumulation of lactate to 116 % and glycerol to 190 % was observed. L/P ratio was elevated indicating ischemia.

In the non-tourniquet group the metabolite changes were less profound and normalized within 60 min.

**Conclusions:**

Microdialysis revealed that performing TKA with tourniquet is associated with increased ischemia. This affects all metabolites but the changes are normalized after 5 h

## Background

In elective TKA the intraoperative use of pneumatic tourniquet is commonly used to minimize blood loss and enhance surgical overview. Despite knowing, that tourniquet induces ischemia and soft tissue damage surgeons still uses it, often not aware of the effects of the induced ischemia [1].

The kinetics of ischemic metabolites during periods of ischemia and reperfusion remains uncertain. The tourniquet pressure combined with ischemia has been investigated and inflicts a more profound damage to the skeletal muscle than ischemia alone [2]. The clinical aspects regarding tourniquet use has been vigorously investigated and cases of rhabdomyolysis have been described [1, 3–6].

The skeletal muscle in limbs is very sensitive to ischemic changes and a clinical assessment is not sufficient to evaluate the degree of ischemic tissue damage induced by the tourniquet [7, 8]. Thus, using an in vivo technique could provide a more accurate assessment of the metabolic events.

Microdialysis (MD) is a minimally invasive technique that allows continuous in vivo monitoring of metabolism in extracellular space [9]. It was originally described by Ungersted and Pycock to monitor neurochemical changes [10]. Interstitial levels of metabolites as glucose, lactate, pyruvate, lactate/pyruvate (L/P) ratio were measured, because they serve as direct indicators of ischemia, whereas glycerol reflects cell damage [8, 11, 12]. During ischemia, lactate increases and pyruvate decreases, leading to an increased L/P ratio, where a ratio above 25 is considered abnormal [13]. Lactate/pyruvate ratio was calculated since it is a precise marker of ischemia and increase during ischemia [14].

The microdialysis catheter consists of a double-lumen linear tube that at the tip has a semipermeable membrane, the tube mimics the functions of a capillary blood vessel. The catheter is connected to a pump that, with a constant flow, pumps the fluid so it can pass the membrane. Diffusion along the concentration gradient occurs in the interstitial space and equilibrium takes place between the fluid and molecules. The molecules are collected in small vials, which reflects the composition of the interstitial space fluid and can then be analyzed immediately afterwards. The metabolites of interests have traditionally been pyruvate, glucose, lactate and glycerol.

To our knowledge MD technique has not been applied in a randomized controlled setup to investigate tourniquet induced ischemia during TKA surgery, even though this could contribute to the understanding of the phenomenon.

The aim of this study was to investigate the in vivo degree of ischemia in skeletal muscle with the use of MD during surgery and cuff inflation and during a period of reperfusion to estimate ischemia and cell damage. In particular the limb distally to the cuff was of particular interest and not directly beneath the cuff, where apparent ischemia takes place.

## Methods

This prospective randomized clinical trial was conducted at Aalborg University Hospital, Aalborg, Denmark. A total of 70 primary TKA were performed between January 2011 and January 2012.

**Fig. 1:**
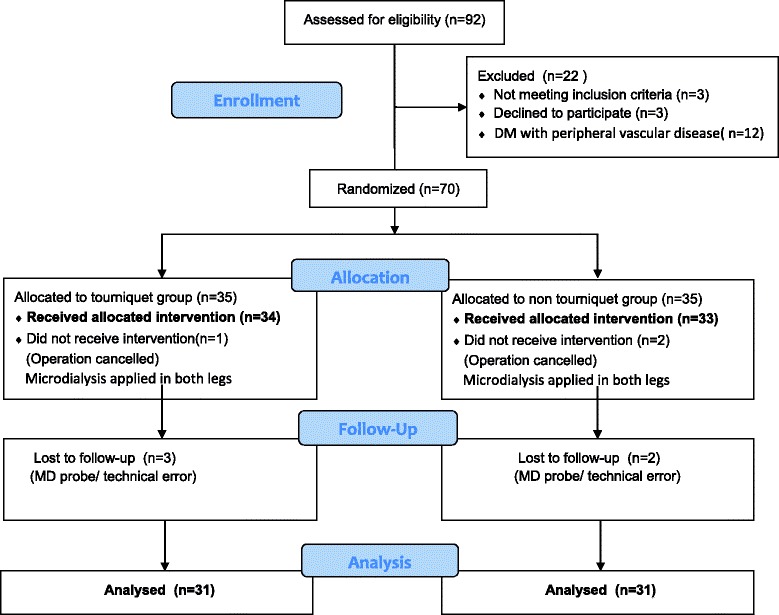
Flow chart of included participants

Approval from the local Ethics Committee (approval no. N-20090045) and registration at ClinicalTrials.gov (NCT01309035) were obtained. All patients gave written consent and were enrolled in this study in accordance with the Consolidated Standards of Reporting Trials (CONSORT) and The Helsinki Declaration (Fig. 1).

This study was part of a larger randomized controlled trial where two other main aims were investigated: tourniquets effect on implant fixation and clinical outcomes. The ischemic conditions of tourniquet use are presented in this publication.

### Patients

Patients aged 50–85 scheduled for primary unilateral TKA were included and were comparable regarding demographics (Table 1). Exclusion criteria included rheumatoid arthritis, peripheral vascular disease, diabetes, prior knee surgery and use of anticoagulation medicine.

**Table 1:** Demographics and perioperative data

	Tourniquet (*n* = 31)		Non tourniquet (*n* = 31)	
	Mean	SD	Mean	SD
Age (yrs)	68,3	8.4	68.2	7.8
Sex m/f	16/15		17/14	
BMI	25.1	2.0	25.2	2.5
Operation time (min)	69.5	5.3	71.3	4.5
Cuff pressure mmHg)	250			
Ischemia duration (min)	74.4	3.7		

Patients were block randomized using sealed envelopes and were allocated into two groups: 34 patients had surgery using a tourniquet (Tq group) and 33 patients had surgery without the use of a tourniquet (non-Tq group). The envelopes were opened when the surgeon was present in the operating theatre before surgery. Patients were unaware of the group to which they had been allocated.

### Microdialysis

In this study we used CMA 60 (CMA Microdialysis AB, Sweden) catheters (length 30 mm, outer diameter 0.6 mm and molecular cut off 20 kDa) in skeletal muscle of the lower extremity. In both groups 2–3 ml lidocaine was injected subcutaneously in the gastrocnemius muscles (vastus medialis) before catheters were inserted parallel to the muscle fibers at an angle of 35°. The correct position of the catheter was verified by ultrasonography. In the non operated leg a catheter was inserted at same level, serving as a reference.

Catheters were connected to a syringe filled with 4 ml perfusion fluid T1 (CMA Microdialysis AB, Sweden) that was placed in CMA 106 MD pumps, that were constant perfused at a rate 0.3 μl/min. Afterwards a period of 40 min of flushing and stabilization was allowed.

The ISCUS MD analyzer (CMA Microdialysis AB, Sweden) with Reagent Set A was used to analyze all the collected MD samples and this was done immediately after sampling.

Before surgery, MD catheters were inserted and the average of the first consecutive samples before performing surgery, were used to establish a baseline and defined as 100 % for metabolites (Table 2). Baseline was measured, after an initial 40 min flushing period followed by a stabilization period of 20 min. In the reference leg, baseline reached stable values within that period of time, and remained unchanged for the whole period of 300 min.

**Table 2:** Average interstitial baseline concentration at a constant flow rate of 0.3 μl/min

Glucose (mmol/L)	5.0 ± 2.0
Pyruvate (μmol/L)	64.9 ± 10.4
Lactate (mmol/L)	1.8 ± 0.4
Glycerol (μmol/L)	84.5 +/12.6
L/P ratio	28.3 ± 3

Immediately after surgery and tourniquet release if that was used, the first sample was collected which defined the time zero (t = 0). The dialysates were regularly collected every 20 min during a 5 h postoperative period, representing time of reperfusion. Samples from each patient were obtained and the changes in the operated leg was compared to the baseline and the patient’s own reference leg. In addition, the non-tq group served as a reference group. Differences between the two groups were compared to evaluate tourniquet effects.

Longer periods of ischemia leave the cells depleted of energy and the ability to regenerate metabolites decreases. The skeletal muscle must change from oxidative phosphorylation to anaerobic glycolysis to create energy and maintain homeostasis. Thus, a rise is seen in lactate production and decrease in glucose and pyruvate [15]. Glycerol is mainly derived from the degradation of phospholipids in cell membrane and increases due to cell damage.

The ratio between the concentration of a metabolite in a dialysate and interstitial space is expressed as “relative recovery”. Recovery of a given dialysate is affected by numerous factors such as molecular weight, surface area of membrane (length and diameter), perfusion flow rate, diffusion rate [9, 16]. Previous studies have described that relative recovery is inversely related to perfusion flow rate, i.e at a slow rate of nearly zero (0.33 μl/min) the relative recovery will be approaching 100 % [9]. The metabolite recovery in present study was defined as 100 %, as relative recovery was not investigated. In the literature most of the experiments are performed with incomplete recovery [8, 12, 15].

### Surgical technique

All procedures were standardized with regard to spinal anesthesia, operative technique and postoperative pain treatment and rehabilitation regimen.

Both groups had an appropriately sized thigh tourniquet applied, but it was only inflated in Tq group. In the non-Tq group, it was placed on the thigh but not inflated, thereby serving as a safety device if uncontrollable bleeding should occur. In Tq group limb exsanguination was done by elevation for 2 min, the cuff was inflated to 250 mmHg. The cuff was not removed until the wound was closed and dressed.

All knee implants were the *NexGen*® CR-Flex Fixed Bearing Knee (Zimmer, Warsaw, Indiana, USA) with use of Biomet Refobacin® Bone Cement R (Biomet, Warsaw, Indiana, USA). In all cases, the patella was resurfaced. Surgical procedures were all performed by the same surgeon. A two-stage cementation procedure was performed. The tibia and patella were implanted first, and then another package of cement was used to fixate the femoral component. This procedure, although more time consuming, was done to secure a careful cementation. Immediately after wound closure, dressings were applied, and the cuff was deflated in the Tq group and removed.

### Statistical analysis

Data for each metabolite over time in each group were analyzed by using analysis of variance (ANOVA), Student’s *t*-test for comparison of the Tq group with the non-Tq group, and Wilcoxon rank sum test if assumption for *t*-test was not fulfilled. Data is presented as mean and standard deviation for normal distributed data. The metabolic changes during surgery and reperfusion are expressed in percentages of baseline values.

The level of significance was set at 95 % confidence limit and *P*-value less than 0.05 was considered significant. Statistical analysis was performed by using STATA 11.0

## Results

Seventy patients were enrolled in the trial: 62 patients (33 male and 29 female) completed the study (Fig 1). Preoperative demographics were similar between groups and showed no significant differences regarding age, weight or gender. The duration of ischemia was 74.4 ± 3.7 min in the tourniquet group.

### Comparison between Tq group and non-Tq group

Comparing the Tq group with non-Tq group differences were registered in all of the metabolites from beginning of reperfusion time and until a period of 180 min. After that there were no difference and the metabolites were restored back to initial levels.

**Fig. 2:**
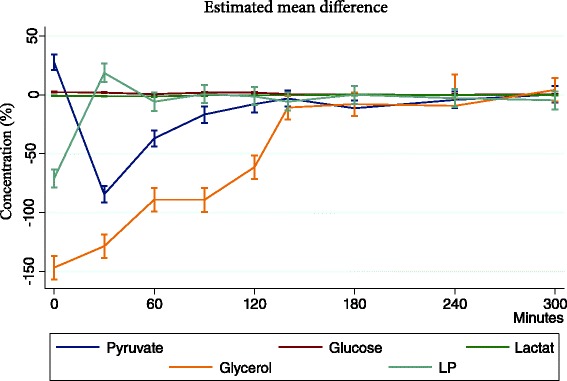
Tourniquet use induces significant ischemia and differences between the Tq group and the non-Tq group are detected in all of the metabolites from the beginning of reperfusion and until 180 min

This is expressed in Fig. 2 where the mean differences between the two groups are shown.

### Tourniquet group

**Fig. 3:**
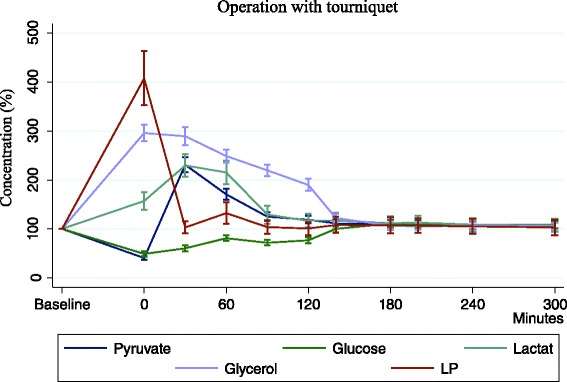
Tq group: absolute values in percentile change from baseline. The ischemic changes are normalized after 300 min

All metabolite concentrations were affected following tourniquet induced ischemia (Fig. 3).

Glucose decreased by 54 % (2.3 ± 0.7 mmol/L; *p* < 0.001) and this reduction was detectable during time of reperfusion, but restored back to baseline 300 min postoperatively.

Pyruvate concentration was initially reduced to 60 % (25.9 ± 5.6 μmol/L; *p* < 0.001), while it was dramatically elevated during first period of 30–60 min of reperfusion to 123 % (145.6+/10.9 μmol/L; *p* < 0.001). At 180 min it was restored back to baseline and no difference was detected (*p* = 0.118).

Concentration of lactate increased significantly during reperfusion of 30–60 min up to 116 % (3.9 ± 0.8 mmol/L; *p* < 0.001). After 120 min of reperfusion it slowly returned to baseline (*p* = 0.129). After 300 min no significant difference was registered (*p* = 0.952) when comparing to baseline (Fig. 3).

Concentration of glycerol also increased dramatically at beginning of reperfusion to 190 % (244.7 ± 12.5 μmol/L; *p* < 0.001) and stayed significant increased during 180 min of reperfusion (*p* < 0.001). At 300 min there was no significant difference (*p* = 0.634).

L/P ratio increased significantly 79 % (107 ± 33.3; *p* < 0.001) after period of ischemia, but after 90 min of reperfusion initial level was restored.

**Fig. 4:**
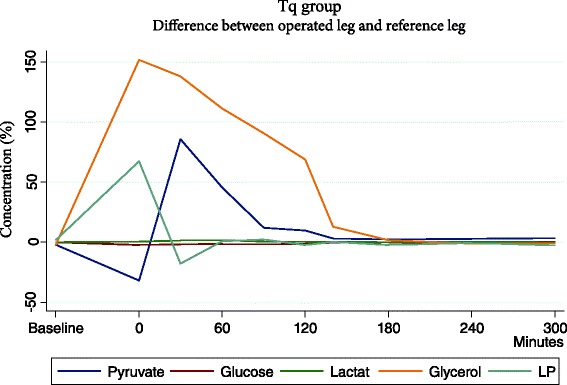
Mean difference between operated leg and reference leg in the Tq group. Significant differences in metabolites are detected until 180 min

Significant differences in all metabolites were noted until 180 min. between operated leg and non operated leg (Fig. 4).

All values returned to baseline values within 300 min in both legs and no difference was registered.

### Non-tourniquet group (non ischemic reference group)

**Fig. 5:**
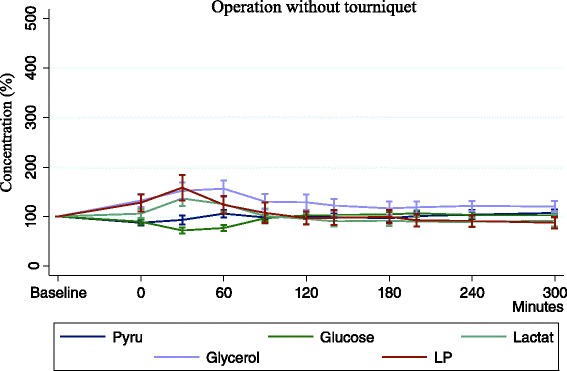
In the non-Tq group metabolite changes were smaller and restored within 60 min

The metabolites were less affected an returned faster back to initial levels (Fig. 5).

The first sample after surgery showed a glucose concentration that only decreased 11.5 % (4.6 ± 0.7 mmol/L; *p* < 0.001) during surgery and during 90 min of reperfusion normal levels were reached (*p* = 0.220).

Pyruvate concentration was reduced to 13.5 % (53.8 ± 9.5 μmol/L) of the initial value and during a short reperfusion period of 30 min, it was back to baseline.

Concentration of lactate increased during early reperfusion and at 30 min it reached a maximum of 30 % (2.6 ± 0.5 mmol/L). After 60 min. it was unaltered and no statistical significance was registered.

Glycerol concentration was increased to maximum of 48 % (114.5 ± 15.4. μmol/L) 60 min postoperatively. During a longer postoperative period it slowly returned to normal (Fig. 5).

**Fig. 6:**
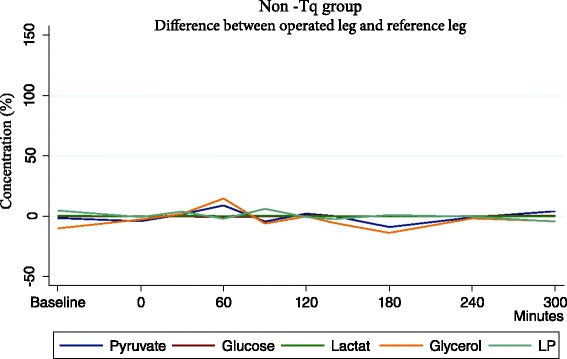
Mean difference in the non-Tq group between operated leg and reference leg. Glycerol is affected due to cell damage as response to surgery. Ischemic metabolites are not affected

L/P ratio also changed significantly reaching maximum at 30 min reperfusion, 45 % (42.2 ± 13.3; *p* < 0.001) but after this time of it was quickly restored. Difference between operated and non operated leg did not show greater ischemic conditions, rather cell damage as a response to surgery was registered, expressed as increase in glycerol (Fig. 6)

## Discussion

Microdialysis is recognized as a useful tool when assessing metabolic changes in skeletal tissue in clinical settings [8, 15]. The present study establishes microdialysis as an effective way to monitor interstitial levels of metabolites during tourniquet induced ischemia. The study was conducted to investigate metabolic changes distally in a limb, since the ischemia underneath the tourniquet has been described previously in literature [15]. The period of interest was when the limb was exposed to ischemia during surgery and the following time of reperfusion. The main findings showed that tourniquet use causes ischemia and cell damage as measured by metabolites. Significant ischemia can be detected in the affected limb until 3 h because of tourniquet use. All Marker levels were normalized after 5 h.

To our knowledge, this study is the first to assess the ischemic changes during TKA surgery caused by tourniquet use in a randomized setup.

The difference between the two groups illustrate that tourniquet application induces changes in level of metabolic markers, which are manifested up to 180 min of reperfusion.

The systemic response to surgery observed in the markers was adjusted for, by using the non-tourniquet group as reference and we concluded that the differences in marker levels are locally affected by the tourniquet induced ischemia.

In the tourniquet group there were significant changes of all the metabolites in the operated leg, when comparing baseline to measurements during reperfusion. The differences were present in all metabolites and were statistically significant comparing from baseline during reperfusion, before slowly returning to normal levels. The non-operated leg, that served as indicator for systemic response, was compared to the operated leg and significant difference were also present at the individual measurement points. The difference between the operated leg and non operated leg represents the local skeletal muscle response in the operated leg.

In the non-tourniquet group, levels of metabolites had changed in the operated leg when comparing baseline to postoperative measurements, but they returned to baseline values faster. Changes were similar to tourniquet group, but not as large or prolonged. When comparing the operated leg and reference leg there was no significant difference, indicating that a local response is not occurring and that the changes are due to an overall systemic response to surgery (Fig. 5).

Thigh pain and swelling has been investigated in other studies, finding tourniquet application being the reason for increased pain and swelling due to ischemia and direct compression [4, 17].

Little is known about basal metabolite concentrations in interstial levels of resting skeletal muscle. Comparing to previous studies our baseline levels are in agreement with data reported earlier [8, 12, 15]. In these studies baseline levels ranged from 3.3–5 mmol/L for glucose, between 1.9 –2.4 mmol/l for lactate, between 40–96 μmol/L for glycerol, while pyruvate was only measured in one study at levels of around 40 μmol/L [15].

Östman et al. [15] used microdialysis to measure tourniquet induced ischemia in patients undergoing arthroscopic surgery. Here measurements were carried out over a time course of 120 min after tourniquet release. All the metabolites were affected and within 120 min the changes were restored back to normal values. The results are in consistence with this present study, where the same metabolite kinetics was observed.

Glycerol is a component of the cell plasma membrane and released into the interstitial space when cells are damaged during surgery. Glycerol can be used as a marker of cell destruction. In addition, high levels of glycerol may also be due to the hormonal regulation of lipolysis and hypoglycemia during tourniquet use which facilitates a catecholamine response that initiate a lipolysis reaction in skeletal muscle [17]. This can partially be a reason for the high rise in glycerol concentration in the tourniquet group, combined with the mechanical compression of the tourniquet.

Increased L/P ratio is a precise marker of ischemia and we observed a difference from baseline immediately after surgery when using tourniquet. After a period of 60 min reperfusion L/P ratio levels were back to normal. In the non-Tq group no difference was observed.

Muscles are believed to be relatively resistant to ischemia, but even shorter or sudden periods of ischemia may result in an overload of calcium in the muscle and could in weaker patients induce secondary complications such as compartment syndrome and respiratory distress syndrome [8, 13]. This should be taken into consideration if tourniquets are applied.

Microdialysis is a minimally invasive technique that has limited risks for patients and even with small concentration volumes, monitoring of the ischemic changes is allowed. It is applicable despite reduced blood flow during ischemia and a continuous sampling of interstitial fluids can be performed. Microdialysis contains a major limitation when estimating data because only an approximation can be stated. Recovery depends upon many factors that affect the equilibrium. To achieve the highest recovery we used the largest membrane recommend to skeletal tissue and the lowest perfusion rate allowed. Most of the clinical studies available use relative recovery and by using very low flow the recovery will be reaching nearly 100 %.

## Conclusions

This study shows that using tourniquet in TKA surgery is associated with increased ischemia during first the postoperative hours but the changes are normalized after 5 h.
